# Genome-wide diversity and gene expression profiling of *Babesia microti* isolates identify polymorphic genes that mediate host-pathogen interactions

**DOI:** 10.1038/srep35284

**Published:** 2016-10-18

**Authors:** Joana C. Silva, Emmanuel Cornillot, Carrie McCracken, Sahar Usmani-Brown, Ankit Dwivedi, Olukemi O. Ifeonu, Jonathan Crabtree, Hanzel T. Gotia, Azan Z. Virji, Christelle Reynes, Jacques Colinge, Vidya Kumar, Lauren Lawres, Joseph E. Pazzi, Jozelyn V. Pablo, Chris Hung, Jana Brancato, Priti Kumari, Joshua Orvis, Kyle Tretina, Marcus Chibucos, Sandy Ott, Lisa Sadzewicz, Naomi Sengamalay, Amol C. Shetty, Qi Su, Luke Tallon, Claire M. Fraser, Roger Frutos, Douglas M. Molina, Peter J. Krause, Choukri Ben Mamoun

**Affiliations:** 1 Institute for Genome Sciences, University of Maryland School of Medicine, Baltimore MD 21201 USA; 2 Department of Microbiology and Immunology, University of Maryland School of Medicine, Baltimore MD 21201 USA; 3 Institut de Biologie Computationnelle, IBC, Université de Montpellier, 860 rue St Priest, Bat 5 - CC05019, 34095 Montpellier, Cedex 5, France; 4 Institut de Recherche en Cancérologie de Montpellier, IRCM - INSERM U896 & Université de Montpellier & ICM, Institut régional du Cancer Montpellier, Campus Val d’Aurelle, 34298 Montpellier, Cedex 5 France; 5 Department of Internal Medicine, Section of Infectious Diseases, Yale School of Medicine, 15 York St., New Haven, Connecticut, CT 06520 USA; 6 Yale School of Public Health and Yale School of Medicine, 60 College St., New Haven, Connecticut, CT 06520 USA; 7 Institut de Genomique Fonctionnelle, IGF - CNRS UMR 5203, 141 rue de la cardonille, 34094 Montpellier, Cedex 05, France; 8 Antigen Discovery Inc., Irvine, CA, 92618 USA; 9 Université de Montpellier, IES, UMR 5214, 860 rue de St Priest, Bt5, 34095 Montpellier, France; 10 CIRAD, UMR 17, Cirad-Ird, TA-A17/G, Campus International de Baillarguet, 34398 Montpellier, France

## Abstract

*Babesia microti*, a tick-transmitted, intraerythrocytic protozoan parasite circulating mainly among small mammals, is the primary cause of human babesiosis. While most cases are transmitted by *Ixodes* ticks, the disease may also be transmitted through blood transfusion and perinatally. A comprehensive analysis of genome composition, genetic diversity, and gene expression profiling of seven *B. microti* isolates revealed that genetic variation in isolates from the Northeast United States is almost exclusively associated with genes encoding the surface proteome and secretome of the parasite. Furthermore, we found that polymorphism is restricted to a small number of genes, which are highly expressed during infection. In order to identify pathogen-encoded factors involved in host-parasite interactions, we screened a proteome array comprised of 174 *B. microti* proteins, including several predicted members of the parasite secretome. Using this immuno-proteomic approach we identified several novel antigens that trigger strong host immune responses during the onset of infection. The genomic and immunological data presented herein provide the first insights into the determinants of *B. microti* interaction with its mammalian hosts and their relevance for understanding the selective pressures acting on parasite evolution.

*Babesia microti*, the primary etiologic agent of human babesiosis, is an emerging health threat worldwide and particularly in the United States. It circulates in a tick vector – mammalian reservoir host cycle, with humans as dead-end hosts. Transmission to humans is primarily effected by ticks in the genus *Ixodes*, but can also occur through blood transfusion and, rarely, through transplacental transmission^1^. The first documented case of human babesiosis attributed to *B. microti* in the United States was reported in Nantucket Island, MA in 1969^2^. Over the past decade, there has been a significant increase in the number of babesiosis cases among both immunocompromised and immunocompetent patients^1^,^2^,^3^,^4^. Patients with asplenia, HIV infection, cancer, hemoglobinopathy, organ transplantation, or those on immunosuppressive drugs or who acquire the infection through blood transfusion, manifest particularly severe disease, sometimes requiring hospital admission and occasionally ending in death or prolonged relapsing illness^1^,^4^.

Current therapies for the treatment of human babesiosis consist of combinations of atovaquone plus azithromycin or quinine plus clindamycin^1^,^5^. Although these drugs have been extensively used in recent years, quinine and clindamycin combination is associated with major side effects, and drug failure has been reported with atovaquone and azithromycin. In some cases treatment can be achieved with higher drug doses, longer treatment duration and/or exchange transfusion, while in others the use of alternative drugs is needed due to microbial resistance^3^,^5^,^6^,^7^,^8^,^9^,^10^. Furthermore, the mechanism by which these drugs exert their anti-*Babesia* activity has only recently begun to be elucidated. Recent studies using a short-term *in vitro* culture as well as immunocompromised mice have shown that, of the four drugs used for treatment of human babesiosis, only atovaquone shows efficacy against the parasite in mouse red blood cells both *in vitro* and *in vivo*^11^. These results, along with the shortcomings of available diagnostic tools to distinguish between past and active infection to prevent transfusion-transmitted babesiosis, have stimulated efforts to improve therapies and diagnostics^11^,^12^,^13^,^14^,^15^,^16^,^17^,^18^,^19^.

There is limited knowledge of *B. microti* diversity in the context of pathogenesis and host-pathogen interactions^20^,^21^,^22^. Similarly, it is uncertain how parasite variability and host adaptation may impact its virulence, its successful transmission to humans, and disease diagnosis and therapy. The paucity of information about this parasite is due in part to the lack of data on genetic variation among isolates, lack of a continuous *in vitro* culture system for propagation of the parasite in human or mouse red blood cells, as well as the absence of tools and resources to manipulate its genome in order to characterize gene function in microbial development and virulence.

Efforts aimed at understanding *B. microti* population diversity and structure, and differentiate between parasite genotypes, have in the past relied on the use of PCR amplification of the 18S rDNA, β-tubulin and the chaperonin-containing t-complex polypeptide 1 (CCT7) (reviewed in ref. 23). More recently, Goethert and colleagues used a genotyping approach based on variable number of tandem repeat loci and identified at least two major populations and shed new light on the mode of expansion of the parasite in southern New England^24^.

The first *B. microti* genome sequencing and analysis were conducted on an isolate named the R1 strain, which provided initial information about genome composition, structure, metabolism, and the phylogenetic placement of the species^25^,^26^. The availability of new sequencing technologies has made it possible to perform genome-wide profiling of genetic polymorphisms in a large number of species including several protozoan parasites^27^,^28^,^29^,^30^,^31^. These analyses have significantly improved our understanding of the diversity and evolution of these pathogens, provided insights into their virulence, host modulation and the genetic basis of drug resistance phenotypes, and helped to develop novel approaches for disease control and prevention, and informed public health policy^32^,^33^,^34^,^35^,^36^,^37^.

Herein we report a systematic and comprehensive study of the genetic and transcriptional diversity of seven *B. microti* isolates. Using data from genomic and transcriptomic analyses, we have re-annotated the entire genome of the reference *B. microti* R1 strain, characterized the genomic diversity among isolates, and identified the full complement of genes encoding the parasite’s secretome and surface proteome. We show that polymorphism in genes that encode secreted and surface proteins accounts for most of the variations found among the *B. microti* isolates analyzed. We generated and screened a protein array consisting of 174 *B. microti* proteins using sera from mice infected with *B. microti* and sera of uninfected mouse controls. We identified 30 new parasite antigens that trigger a strong host response and determined that some of these antigens are encoded by the genes that are most polymorphic among these isolates. The fact that the most highly expressed genes in *B. microti* are unique to this species suggests that it has evolved novel mechanisms for survival within human red blood cells and to interact with its mammalian host. The genetic patterns reported here are valuable tools for a better understanding of *B. microti* pathogenesis and mode of transmission, and may contribute to improve disease diagnosis and control.

## Results

### Genome variability among *B. microti* isolates

**Figure 1:**
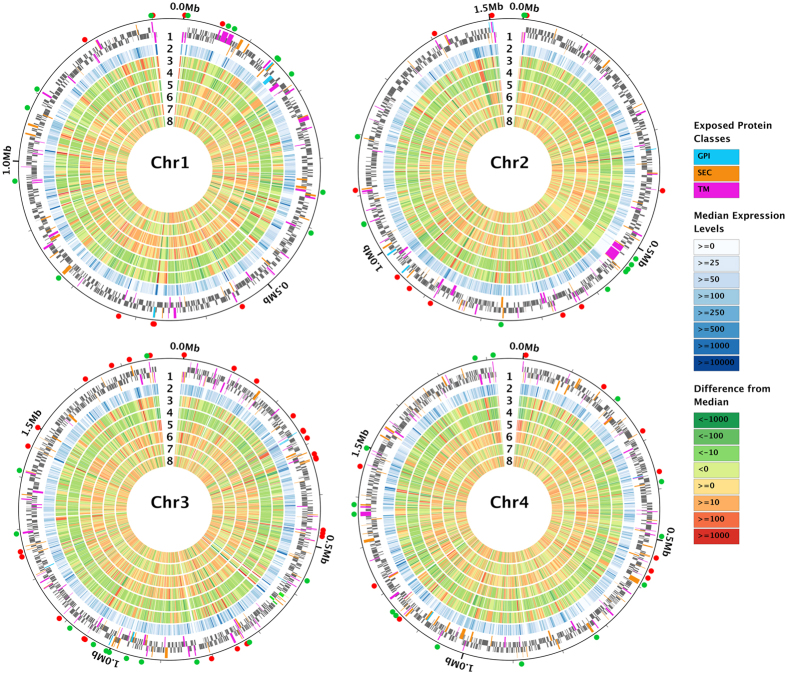
Gene expression profile is highly correlated among six isolates of *B. microti*. Gene expression is shown for genes across the four nuclear chromosomes of *B. microti*. The numbered concentric circles represent the following: **1**) genes encoded in the forward and reverse strands (mostly grey), with the host-exposed, secretome genes color-coded (GPI-anchored in blue; secreted proteins, SEC, in orange; transmembrane proteins, TM, in pink), and rDNA genes highlighted (in green, in chromosome 3); (**2**) median gene expression level, in RPKM (shades of blue); (**3–8**) deviation from median expression level, respectively, in GI, ATCC 30222, ATCC PRA-99, GreenwichYale_Lab_Strain_1, Nan_Hs_2011_N11_50, and Naushon. The expression of most genes is very similar across isolates, as shown by the overall low color intensity in these six most inner circles, with some notable exceptions such as the subtelomeric transmembrane-encoding genes in chromosome 1, or the GPI-anchored proteins, shown at 6 o’clock, in chromosome 1. The secretome genes that fall among the 10% most highly expressed (red) and 10% least expressed (green) genes are marked with small circles outside each chromosome plot. Approximately 25% of the secretome genes fall into one of these two classes.

To advance our understanding of *B. microti* genetic diversity and adaptation to the host environment, we performed high-throughput, whole genome sequencing of seven *B. microti* isolates (Supplementary Table S1), re-sequenced the reference R1 strain, and generated RNAseq data from six of these isolates (Supplementary Table S2). All nucleic acid material was obtained from the intra-erythrocytic life cycle stage of parasites propagated in rodents. Nearly all (98.3%) of the 3,567 protein-coding genes in the *B. microti* genome were found to be expressed, as defined by an average RPKM ≥ 10 and ≥4X coverage. Our analyses revealed that gene expression is highly correlated among isolates (0.81 < *r* < 0.94; Fig. 1), with intron-exon splice sites clearly defined in the vast majority of genes (http://jbrowse.igs.umaryland.edu/b_microti). This information provided exceptional transcript resolution and made possible extensive manual curation and validation of the structure of nearly all protein-coding genes, resulting in improved structural characterization of 52% of all these genes (Supplementary Tables S3–S5). The updated nuclear genome annotation consists of 3,615 genes, 3,567 of which encode proteins, making it one of the most gene-dense genomes identified so far among the Apicomplexa (Supplementary Table S3). The updated re-annotation identified 70 new genes, with changes to previously predicted genes being relatively minor. Based on RNAseq analysis, ~64% of all exons in the new annotation were correctly predicted in the original annotation and ~34% were re-annotated. Only 4% of all nucleotides that are part of protein-coding sequences had been incorrectly assigned to introns or intergenic regions in the original annotation (Supplementary Table S4). Remarkably, *B. microti* genes are characterized by a preponderance of unusually small introns ranging in size between 18 and 21 nucleotides (some of which are in frame), a rare occurrence among eukaryotes (Supplementary Fig. S1). We also performed deep sequencing of the *B. microti* R1 isolate in order to validate the sequence of the reference genome. Only 36 differences were found (Tables 1 and 2), mostly associated with chromosome ends (78%). Of these, 35 are insertions/deletions (indels), with 25 being in intergenic regions. Nine of these differences (one SNP and eight indels) may correspond to sequencing errors in the original assembly, as the alternate variant was found not only in the R1 re-sequencing data but also in all seven newly sequenced *B. microti* isolates. The remaining 27 differences either also correspond to sequencing errors in the original assembly or else may have accumulated during passaging of the R1 isolate in gerbils.

**Figure 2:**
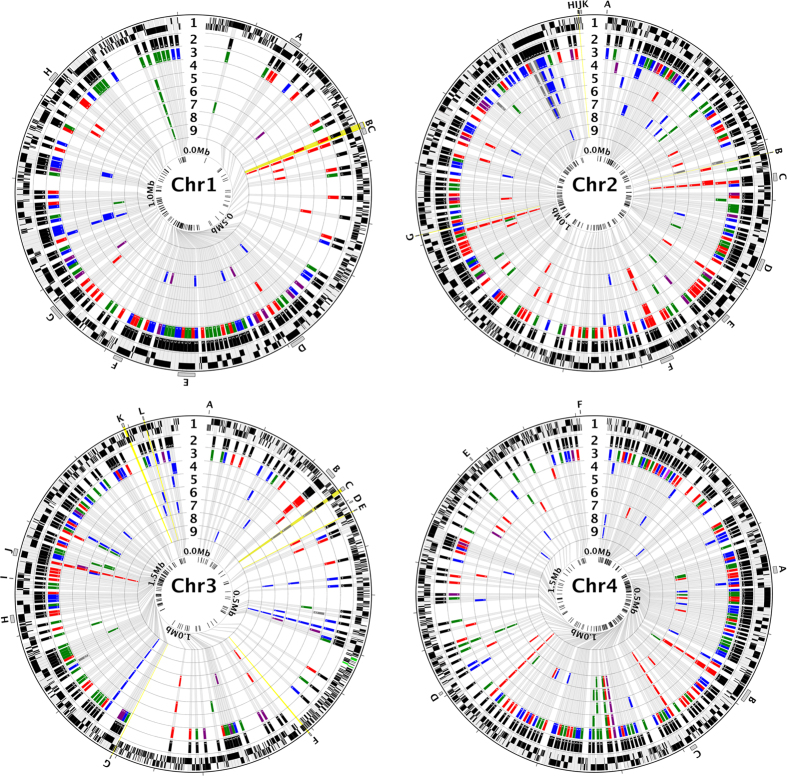
Sequence variants are rare among eight *B. microti* isolates. Distribution of all SNPs relative to GI strain, across the variable genes in each of the four nuclear chromosomes of *B. microti*. In each chromosome plot, the numbered concentric circles represent: **1**) genes encoded in the forward and reverse strands (black; rDNA genes in green, in chromosome 3), with loci with sequence variants in any of the strains expanded 5000X; (**2**) character state, for each variable site, in the GI strain; (**3–9**) SNPs shown relative to GI, respectively, in R1, Bm1438, ATCC 30222, ATCC PRA-99, GreenwichYale_Lab_Strain_1, Nan_Hs_2011_N11_50, and Naushon. SNP color-code relative to GI: non-synonymous in red; synonymous in green; UTR SNPs in purple; intron and intergenic SNPs in blue. The inner-most circle shows the location of variable loci, with the genomic position shown to scale. The vast majority of SNPs are fixed differences in R1 (track 3), relative to all other strains. Loci that encode the ten most immuno-dominant proteins are highlighted (radial yellow, in chromosomes 1–3; letter-coded). Letter-coded loci also include the 27 most polymorphic genes, which together contain nearly 1/3 of all non-synonymous mutations. **Letter code: Chr1: A,** BBM_01g00480; **B,** BBM_01g00985; **C,** BBM_01g00996; **D,** BBM_01g01915; **E,** BBM_01g02065; **F,** BBM_01g02135; **G,** BBM_01g02415; **H,** BBM_01g03070. **Chr2: A,** BBM_02g00010; **B,** BBM_02g00896; **C,** BBM_02g01030 **D,** BBM_02g01290; **E,** BBM_02g01490; **F,** BBM_02g01825; **G,** BBM_02g03140; **H,** BBM_02g04250; **I,** BBM_02g04260; **J,** BBM_02g04265; **K,** BBM_02g04280. **Chr3: A,** BBM_03g00006; **B,** BBM_03g00690; **C,** BBM_03g00785; **D,** BBM_03g00885; **E,** BBM_03g00947; **F,** BBM_03g02345; **G,** BBM_03g03430; **H,** BBM_03g03985; **I,** BBM_03g04060; **J,** BBM_03g04135; **K,** BBM_03g04695; **L,** BBM_03g04760. **Chr4: A,** BBM_04g05770; **B,** BBM_04g06095; **C,** BBM_04g06375; **D,** BBM_04g07535; **E,** BBM_04g09150; **F**, BBM_04g09980.

**Figure 3:**
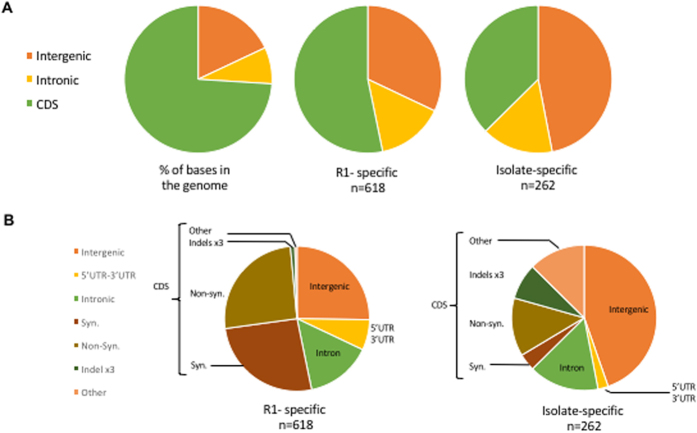
Comparative analysis of mutation frequency among *B. microti* isolates. (**A**) Pie charts illustrating the mutation frequency in coding sequences (CDS), introns and intergenic regions as a percentage of total base pairs in the genome (left) and as a percentage of all mutations found to be specific to R1 (middle) or found in other isolates but not R1 (right). (**B**) Pie charts illustrating the details of the mutation frequency in genes in R1 (left) and other isolates (right).

Analysis of genetic variation among genomes revealed a remarkable dearth of genetic diversity (Tables 1 and 2, Fig. 2), despite the fact that the isolates were collected from different geographic areas, across several decades, and represent recent clinical infections as well as long-established lab strains (Supplementary Table S1). A total of 889 variable positions, defined by either single nucleotide polymorphisms (SNPs) or short indels, were found in the 6,395,281 bp *B. microti* R1 genome assembly (Tables 1 and 2, Supplementary Table S8). The average pairwise difference between each isolate and the reference R1 is 588 SNPs (Table 1), corresponding to 0.9 SNPs/10Kb, a frequency over one order of magnitude lower than current SNP density estimates for the human malaria parasite *Plasmodium falciparum*^38^. The majority of the variable positions were found to be R1-specific, with 515 SNPs and 103 indels unique to this isolate (Tables 1 and 2, Supplementary Table S7, and Fig. 3A). On the other hand, only 262 variable sites (with SNP or indels) were found among the other seven genomes, with an average of 14 mutations being unique to each isolate, and 150 shared by two or more isolates (Supplementary Table S6). SNPs were enriched in non-coding segments (Fig. 3B), which correspond to 26% of the genome but accumulated ~38% of all SNPs (Chi-square, *P* < 0.0001). The distribution of indels was even more skewed, with only 20% of all indels found in coding sequences (CDSs) and nearly half of them result in in-frame mutations. Of the 3568 protein-coding genes, only 205 carried a combined total of 257 amino acid-altering mutations, including indels of length not a multiple of three, and non-synonymous, read-through or non-sense SNPs.

**Figure 4:**
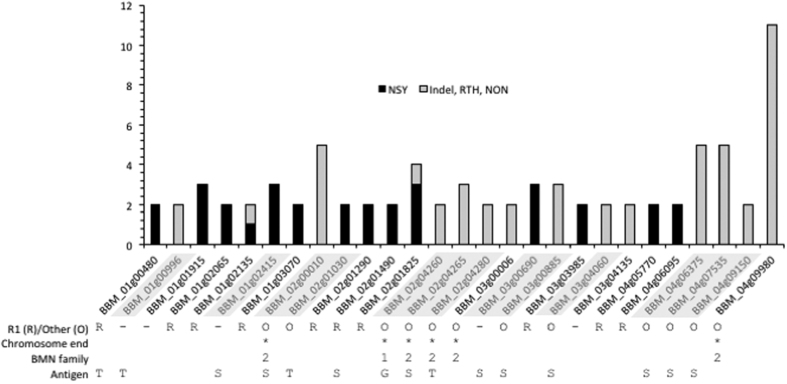
Distribution of non-synonymous (NSY: black), and indels, read-through (RTH) and non-sense (NON) (light gray) mutations in *B. microti* coding sequences with at least 2 mutations. Genes carrying R1- specific mutations are indicated with (R), those carrying non-R1specific mutations are indicated with (O), and those genes carrying both R1 and non-R1 specific mutations are labeled with (−). Genes located in chromosome ends are marked with (*). Genes encoding members of the BMN family are marked with (1) to indicate the BMN1 family and (2) for the BMN2 family. Antigens depicted are GPI-anchored protein (G), transmembrane proteins (T) and secreted proteins (S). Genes encoding members of the secretome with non-R1 specific mutations are highlighted in gray.

**Figure 5:**
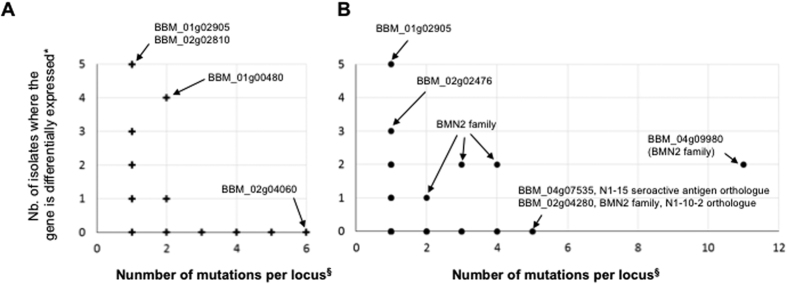
Comparison of expression levels of genes carrying at least one mutation. *Number of isolates where gene expression shows a 3-fold difference from the median of the six tested isolates. ^§^Number of mutations found in CDS, intron, 5′ UTR and 3′ UTR. (**A**) Loci with R1-specific mutations (n=383). (**B**) Loci with non-R1-specific mutations (n=105).

**Figure 6:**
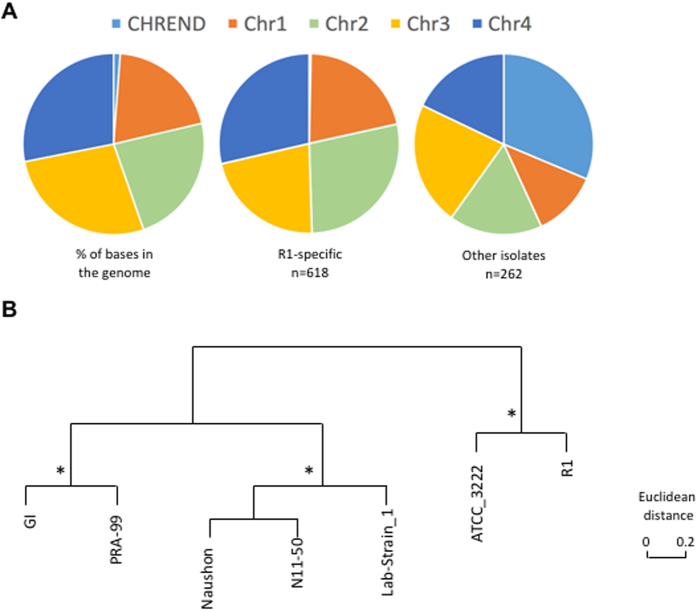
Comparative analysis of mutation frequency in chromosome ends of *B. microti* isolates. (**A**) Pie charts illustrating the mutation frequency in chromosome ends and coding core of chromosomes I to IV as a percentage of total base pairs in the genome (left), and as a percentage of all mutations found to be specific to R1 (middle) or those found to be isolate specific (right). (**B**) Clustering analysis of *B. microti* isolates based on 12 microsatellite markers. *Conserved nodes observed in clustering based on differentially expressed genes.

Twenty seven genes contain nearly one third of all non-synonymous mutations (31 SNPs) and indels (48 indels) (Figs 2 and 4). More than half of these 27 genes encode surface or secreted antigens, including five members of the *BMN2* gene family, which accumulated 23 of the 257 non-synonymous mutations (Figs 2 and 4). The most variable gene identified in this analysis is BBM_04g09980, with 11 mutations, and is located in the sub-telomeric region of chromosome IV. This gene is also differentially expressed between isolates (Fig. 5). Interestingly, the intergenic regions flanking BBM_04g09980 are also highly polymorphic. Overall, our analyses revealed that chromosome ends account for 9.3% of genome variations observed in the genome of *B. microti* (Fig. 6A). The genes in this region of the chromosomes are variable between strains (Fig. 6A) and are associated with the presence of indels. SNPs in these regions were often below the quality threshold applied for calling because of the presence of several sequences that are repeated multiple times. Sequencing of PCR products confirmed the scarcity of SNPs at chromosome ends.

Analysis of microsatellites in the genome of the *B. microti* isolates revealed 336 micro- and mini-satellites ranging in length between 2 and 375 bp. Among these, 12 are variable among strains with 8 found in coding sequences, 3 in intergenic regions, including previously described BMV4^24^, and 1 in an intron. Clustering of the seven isolates based on these microsatellites showed that they form three major clusters one comprised of G1 and PRA-99, the second of Naushon, N11–50 and GreenwichYale_Lab_Strain_1 (LabS1), with the ATCC-30222 and the R1 isolate forming a sister group to the other isolates (Fig. 6B).

### *Identification of B. microti* genes encoding components of the secretome and potentially under immune selection

**Figure 7:**
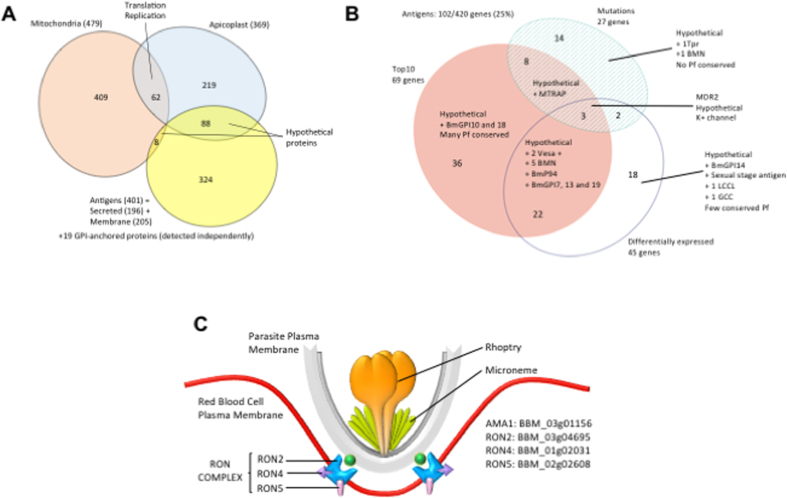
*B. microti* host-presented antigen dataset. (**A**) Venn diagram illustrating the composition of the *B. microti* set of proteins targeted to the mitochondria, apicoplast and the secretome. Identification of the mitochondrial and apicoplast sets of proteins helped curate the main constituents of the secretome, from which the organelle-targeted genes were excluded. (**B**) Overlap between secretome-encoding genes and genes that either harbor SNPs and indels, are ranked in the top 10 most expressed *B. microti* genes, or are differentially expressed between isolates. (**C**) Model of the moving junction core complex of *B. microti* predicted based on the updated genome annotation.

To identify proteins of *B. microti* that might play a role in host-parasite interaction and immune modulation, we curated the proteome for all possible members of the *B. microti* secretome, including GPI-anchored, secreted and transmembrane proteins, based on primary sequence attributes as well as on homology to members of the malarial secretome (Supplementary Table S8). *B. microti* proteins predicted to localize to intracellular organelles were excluded from this set (Fig. 7). The *B. microti* secretome consists of 420 proteins, 19 of which are GPI-anchored (GPI), 196 are predicted to be soluble secreted proteins (SEC) and 205 associated with a membrane (TM). This set encompasses all previously described antigens such as the BmP94 antigen (BBM_04g08155), the maltese-cross seroactive antigen (BBM_04g07535), most of the BMN genes, and several small multigene families (Tpr, Vesa, Rhomboid, CRMP, PSOP, LCCL and CPW-WPC)^26^,^39^,^40^,^41^,^42^,^43^,^44^,^45^. More than half of the genes encoding components of the *B. microti* secretome are unique to this species. 299 of which encode hypothetical proteins, including 82 that have homologs in *P. falciparum*. Whereas approximately 60% of all *B. microti* proteins have homologs in *P. falciparum*, that proportion is only 36% among the 420 proteins of the *B. microti* secretome^46^,^47^,^48^,^49^,^50^. Conversely, of the >500 *P. falciparum* proteins predicted to be secreted (http://mpmp.huji.ac.il/), only 151 have homologs in *B. microti*. As evidence of major differences between *B. microti* and *P. falciparum* in their interaction with the host cell and its remodeling, none of *P. falciparum* red blood cell (RBC)-targeted proteins are found in *B. microti* (Supplementary Table S8) and no homologs to the components of the *P. falciparum* PTEX translocon are found in this parasite^51^. Furthermore, of all the known microneme and rhoptry proteins of *P. falciparum*, only 16 are found in *B. microti*, including nine homologs of rhoptry-associated proteins (BmRAP1–9), homologs of components of the moving junction BmAMA1, BmRON2, BmRON4 and BmRON5, two homologs of the rhoptry bulb constituents (BmRhopH2 and BmARO) and another microneme protein BmPLP1^52^ (Fig. 7C). Interestingly, a homolog of the endoplasmic reticulum protease Plasmepsin V (BBM_04g05270), proposed to play a role in the processing of some secreted proteins in *P. falciparum*^53^, is found in the *B. microti* proteome, albeit with little sequence homology in the C-terminus part of the aspartyl protease A1 domain (Supplementary Fig. 2). No protein homologs of the targets of Plasmepsin V in *P. falciparum*^46^,^48^,^49^, *T. gondii*^54^ or *B. bovis*^55^ could be found in *B. microti*. New studies are needed to identify possible targets of the Plasmespin V–like peptidase from *B. microti*.

RNAseq analysis showed that while some of the genes encoding members of the *B. microti* secretome are highly expressed during blood stage infection, others are either not expressed or expressed at very low levels during this phase of the parasite life cycle (Fig. 1). Members of the sub-telomeric multigene families, including the *Tpr*-like genes, are expressed but at different levels, suggesting that they are independently regulated.

### Differential gene expression among *B. microti* isolates

**Figure 8:**
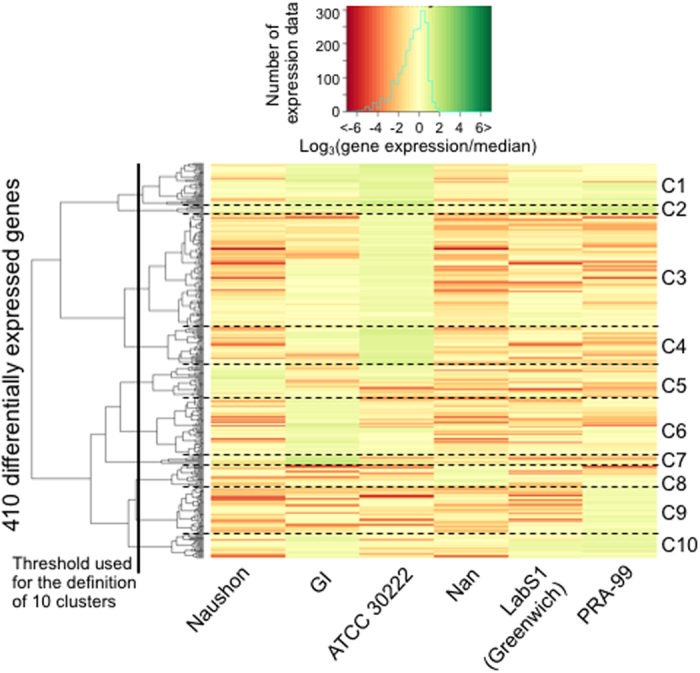
Heatmap of the log-transformed relative expressions of 410 genes considered as differentially expressed among 6 *B. microti* isolates. The gene expression was characterized as the log_3_ of the ratio between expression level in a specific isolate over the median of the expression levels in the 6 isolates. The distance between genes is the Euclidean distance. Ward metrics was used to obtain the dendrogram. Ten gene clusters could be characterized after classification.

Transcriptional analysis showed a few major differences in expression levels among *B. microti* genes, with secretome protein classes being among the most variable (Figs 1 and 8A–I). The three most expressed genes in *B. microti* in both mice and hamsters are those encoding the GPI-anchored proteins BmGPI12 and BmGPI13 and the sugar:H+ symporter BmHT1. Comparison of gene expression between different isolates shows that the vast majority of the genes are similarly expressed in all isolates, with correlation of gene expression between each pair of isolates ranging from 81% and 94%. However, there are some noticeable exceptions, with 410 genes (including 33 rRNA and tRNA genes) that are differentially expressed among strains (defined as RPKM differing by more than 3 fold from the median RPKM; Supplementary Table S8), with differences between isolates surpassing 30 fold. The threshold was benchmarked using several housekeeping genes including the 18S rDNA, and the genes encoding *B. microti* translation elongation factor EF1α and EF1β, glyceraldehyde-3-phosphate dehydrogenase, succinate dehydrogenase subunits and lactate dehydrogenase (Fig. 8A–C). Thirty nine genes showed differential expression with levels of expression at least 10X higher or lower than the median. These include members of the putative parasite secretome as well as a neck kinase 4 ortholog (BmNEK4: Bm_03g00715), which was highly expressed only in the *B. microti* ATCC-30222 isolate (Fig. 8F). Other genes showed differential expression in at least 2 isolates, and include six encoding hypothetical proteins and members of the parasite secretome.

Different *B. microti* isolates showed different host specificity, and therefore we have also compared host-dependent expression differences between isolates. Using EdgeR and DEseq2 methods to correlate gene expression to host specificity, 59 genes were identified in both analyses (Fig. 8G–I); 47 were up-regulated in isolates grown in hamsters, and 12 genes were up-regulated in isolates propagated in SCID mice. Of the 59 genes, 50 were found to be differentially expressed between isolates grown in different hosts with at least 3-fold increase or decrease in expression (Supplementary Fig. 3). Twenty-seven of these genes encode proteins with unknown function, whereas twelve are members of the secretome and six are involved in the regulation of protein expression, including the E3-ubiquitin ligase subunit, elongation factor eIF1B subunit and the mitochondrial subunit S8. Trafficking and cytoskeleton-related functions were attributed to three and two proteins, respectively. The secretome group includes three BmS48/45 genes, encoding the GPI-anchored antigen homolog of the *P. falciparum* sexual stage antigen Pfs48/45, which are highly expressed in parasites grown in hamsters.

The high prevalence of candidate antigen-encoding genes among differentially expressed *B. microti* genes, and the fact that these genes are twice as likely to be polymorphic as other parasite genes, suggest a possible role for these antigens in immune modulation.

### Immunoproteomic analysis of *B. microti* major antigens

**Figure 9:**
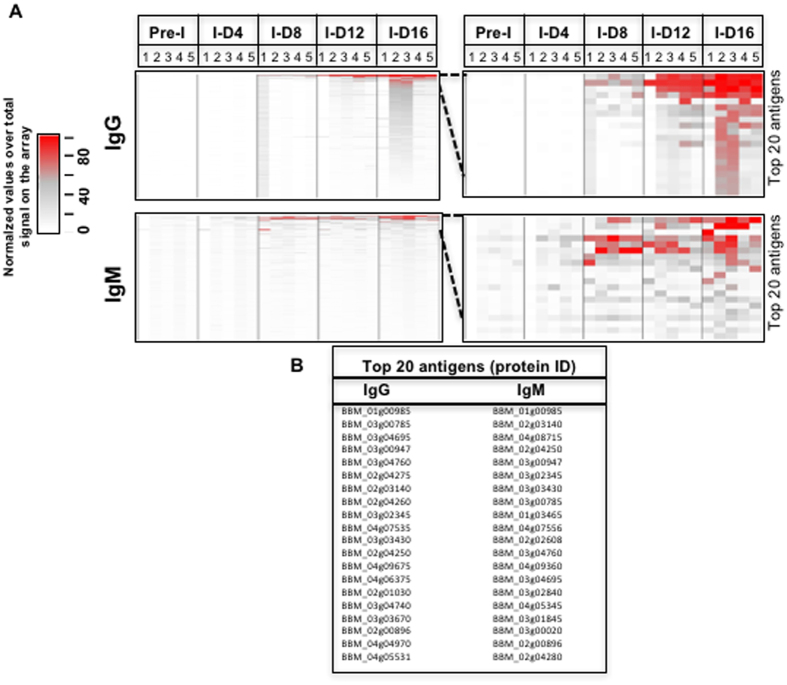
Humoral immune response against the *B. microti* secretome. An antigen down protein array consisting of 198 selected *B. microti* polypeptides corresponding to 174 unique proteins and screened it using pre-immune as well as immune sera collected from wild type Swiss Webster mice at days 4, 8, 12, and 16 following inoculation with *B. microti* LabS1 strain. Heatmaps for all 198 polypeptides were generated with the IgG (**A**) and IgM (**B**) intensity data. The color scale goes from white > grey > red representing low > mid > high serum reactivity to the spots. Each row is a feature/protein on the microarray and each column is a sample. A zoomed in view for the top 20 reactive antigens with row labels was also generated (**C,D**). This same data was used to create a line graph for the top 20 reactive antigens to show binding kinetics for IgG (**E**) and IgM (**F**) antibodies more clearly.

**Figure 10:**
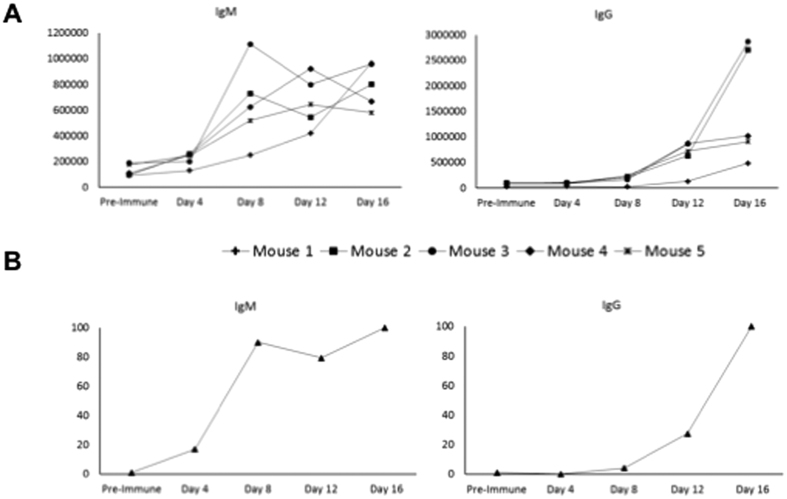
Progression of the IgM and IgG responses in mice overtime as determined by immunoproteomic analysis. (**A)** IgM and IgG signals measured on a protein array for each individual mouse over time. (**B**) Median of the IgM or IgG responses determined for 174 *B. microti* proteins.

In order to identify *B. microti* proteins that trigger a humoral immune response, we developed a reverse phase, antigen down, protein array consisting of 174 predicted proteins. We screened the array using pre-immune as well as immune sera collected from wild type Swiss Webster mice at days 4, 8, 12, and 16 following inoculation with *B. microti* Lab Strain 1. Whereas no antibodies could be detected with naïve, pre-immune sera, analysis of the kinetics of the humoral immune response associated with *B. microti* infection phase identified several new antigens, 62% of which were constituents of the *B. microti* secretome (Figs 9 and 10). Detectable levels of IgM antibodies were measured as early as day 4 post-infection and increased significantly over time, peaking at day 8 and remaining high until day 16 post-infection (Figs 9 and 10). In contrast, IgG antibodies were very low at day 4 and increased over time reaching their peak at day 16 (Figs 9 and 10). The immune signature of the top 20 IgG or IgM most highly antigenic proteins identified 30 proteins (Fig. 9). Nearly half (14/30) are part of the secretome (5 GPI, 6 SEC and 3 TM). Only four genes are polymorphic, with one variable site each (SNP or indel). Interestingly, all US isolates outside R1 encode the same allele in each of those loci. Analysis of the protein array data resulted in the identification of three subsets of 10 proteins each. The first set includes proteins that trigger strong IgM and IgG responses starting at day 4 for the former and at day 8 for the latter, and remain high until day 16. This subset includes BmGPI12/BmSA1 (BBM_01g00985), a secreted S1/P1 nuclease (BBM_02g03140), BmRON2 (BBM_03g04695) and two secreted hypothetical proteins (BBM_01g00985 and BBM_03g00947). With the exception of *BmGPI6* and *BmGPI17* (BBM_02g00896 and BBM_03g03430 respectively), all genes encoding antigens in this subgroup are among the top 10% most expressed genes in *B. microti*. The strongest immunogenic responses were obtained against BBM_01g00985- and BBM_03g00947-encoded peptides, both of which are part of the secretome. Both genes contain non-synonymous polymorphisms (Supplementary Table S8), including a variable microsattelite in BBM_03g00947 which supports the three groupings shown in Fig. 6B. In addition, BBM_03g00947 is downregulated in the Naushon strain relative to the other isolates. The second subset consists of proteins that trigger a significant IgG response that increases over time, and peaks between days 12 and 16, but induced only a moderate to weak IgM response over the 16-day period. Most notable among these are three GPI-anchored proteins (BmGPI9 and 10) and the N1–15 maltese-cross seroactive antigen orthologue (BBM_04g07535). Seven of the proteins in this set are members of the secretome. The third subset includes proteins that trigger a strong immune IgM response and a low to weak IgG response. Half of the antigens in this group are members of the secretome including BmRON5 and two members of the BMN2 family.

## Discussion

In this study we have combined genomic sequencing of seven *B. microti* isolates with transcriptomic analyses to systematically characterize the diversity of this emerging pathogen. Our sequencing of seven new isolates and re-sequencing of the R1 reference genome confirmed the previous genome analysis, which indicated that *B. microti* has the smallest apicomplexan genome available to date, and is among the most gene-dense. Draft genome assemblies generated for the different isolates confirmed a genome size around 6.5 Mb (Table S2), approximately one-fourth the size of *Plasmodium* genomes. Despite the significant difference in genome size, a careful manual curation of gene models, facilitated by RNAseq data, showed the total number of *B. microti*-encoded genes to be 3567, almost two thirds the number in the *P. falciparum* genome, which consists of 5324 genes. The parasite secretome consists of 420 proteins, over 10% of its proteome. Secretion of these proteins to the host membrane or environment to remodel the host cell, acquire nutrients, or modulate the host immune response most likely involve the standard secretion pathway. Our analysis showed that no components of the *Plasmodium* translocon exist in *B. microti*, and that no homologues of proteins secreted through the translocon pathway are found in *B. microti*. Analyses based on sequence similarity failed also to suggest the use of other known secretion pathways, such as those associated with dense granules in *Toxoplasma gondii*^54^ or spherical bodies in *Babesia bovis*^55^. The role of the Plasmepsin V–like peptidase found in *B. microti* remains to be clarified in the absence of large multigene families.

By comparing the sequences of seven new *B. microti* isolates with the genome of the reference R1 isolate, we have identified only a total of close to 900 variable sites, including 588 SNPs and 301 indels. An analysis of the distribution of SNP-associated parameter values for each parameter considered, together with amplicon sequencing-based validation, was critical for the accurate identification of SNPs and in particular the elimination of false positive variants. The extraordinarily low sequence polymorphism found among these isolates, which originate primarily (but not uniquely) from the Northeast region of the United States, suggest that they all share a very recent common ancestor, possibly in the hundreds of years, assuming a mutation rate similar to other eukaryotes^56^. However, this finding needs to be confirmed with a more extensive population survey, accurately identified sequence variants, and coalescent modeling. Quite surprising is the lack of sequence divergence between the isolate ATCC 30222, thought to be originally from Zaire, and the remaining isolates, all of which are believed to originate from the Northeast United States. This issue that might require additional investigation to ensure the provenance of this ATCC isolate.

Three major variation-associated patterns were found among the *B. microti* isolates examined in this study. First, the R1 isolate appears significantly different from all other isolates with R1-specific mutations representing 90% of all microsatellites and nearly 70% of all SNPs and small indels. Re-sequencing of the R1 isolate further validated the uniqueness of the R1 genome. Interestingly, R1 was isolated from a babesiosis patient who likely contracted the disease in Nantucket, MA. It is possible that the R1 isolate represents a different *B. microti* lineage from all other isolates. A recent study by Lemieux and colleagues^56^, released while this article was under review, suggests that all non-R1 isolates sequenced here likely belong to a New England lineage of *B. microti* separate from that containing the R1 reference. Second, non-R1 specific mutations, and differences in gene expression among isolates, are significantly associated with chromosome ends, a pattern similar to the accumulation of new mutations documented in other apicomplexans^57^,^58^. Finally, much of the non-synonymous variation identified among isolates falls disproportionately in a small number of genes, including many members of the secretome, suggestive of immune system-related selective pressure.

Immuno-proteomic analyses show that few members of the secretome induce IgM and/or IgG responses (Fig. 9). Among them, the BmSA1 antigen (BBM_03g00785) has already been placed among the most promising proteins for the development of a detection assay for *B. microti* in blood samples^19^. Two other proteins from the secretome provide even stronger signal by reverse phase analysis in mice: BBM_01g00985 and BBM_03g00947. Combined analysis of the genome-wide variation, transcriptome and immuno-proteome further confirmed the relevance of the GPI-anchored protein set in parasite-host interaction. The GPI-anchored proteome of *B. microti* is composed of only 19 proteins^19^, but six were found to be among the 20 proteins inducing the strongest IgG or IgM responses. We also found that nine of GPI-anchored proteins were among the top ten most highly expressed genes, among the set of genes harboring non-synonymous mutations and/or among the set of differentially expressed genes (Fig. 5). All genes from the BMN2 family are among these proteins. Nine of these were members of the secretome but they show little immunogenicity, suggesting a possible role in antigenic variation.

It remains unknown whether *B. microti* host preference can be linked to specific genetic determinants. However, two new lines of evidence generated in this study support this possibility. First, RNAseq analysis revealed 59 parasite genes with significantly different expression levels between isolates grown in different rodent host systems. In addition, our attempts to propagate these isolates in small rodents revealed clear host preferences.

In conclusion, our genomic and transcriptomic analyses of *B. microti* isolates provides initial evidence that *B. microti* strains from the Northeast region of the U.S. are not highly diverse and that most polymorphisms in this parasite are found in genes encoding proteins likely to be involved in host-pathogen interactions. Several antigens might prove useful in the development of a specific and sensitive assay for rapid detection of *B. microti* infection as well as for antibody-based targeted therapy.

## Material and Methods

### Ethics statement

All animal experimental protocols followed Yale University institutional guidelines for care and use of laboratory animals and were approved by the Institutional Animal Care and Use Committees (IACUC) at Yale University (Protocol #2010-07689). Yale University is accredited by the American Association for Accreditation of Laboratory Animal Care (AAALAC Number 101), and has an approved Animal Welfare Assurance (#A3230-01, effective until 5/31/2011) on file with the NIH Office for Protection from Research Risks. Rules for ending experiments in animals were to be enacted if animals showed any signs of distress or appeared moribund. This, however, was not the case for any animals in the study.

### Animals

CB17/Icr-*Prkdc*^*scid*^/IcrIcoCrl mice and Golder Syrian Hamsters were purchased from Charles River, Inc. Animals were inoculated with infected blood via i.p. injection and monitored for infection. Parasitemia was determined using standard methods for collecting a drop of blood from the tail vein and using this blood to perform Giemsa staining.

### ***B.** microti* Isolates

*Babesia microti* isolates used in this study are: GreenwichYale_Lab_Strain_1 (Lab_Strain_1), a tick isolate propagated in mice and kindly provided by Dr. Durland Fish. Two isolates obtained from BEI Resources: ATCC-30222, and ATCC-PRA99. Two isolates kindly provided by Dr. Sam Telford: GI and Naushon. Two clinical isolates obtained from blood collected from babesiosis patients in 2011 and 2014, respectively: Nan_Hs_2011_N11-50 (N11-50) and Bm1438. These isolates were injected into SCID mice and/or hamsters and infection was monitored for at least 2 months by microscopy analysis of Giemsa-stained blood (Supplemental Table 1).

### Serum collection

Mouse sera were collected as follows. Five 6-week old female Swiss Webster mice were used to collect blood on day 0 (pre-immune sera) prior to infection with the *B. microti* LabS1 strain. Infection was achieved by IP injecting of 10^7^ iRBCs previously collected from an infected SCID mouse. Blood was then collected from the five mice on days 4, 8, 12 and 16 in microcentrifuge tubes and left at room temperature for 3 hours. After centrifugation at 4 °C for 10 minutes at 13,000 rpm, the serum fraction was collected in microcentrifuge tubes and stored at −80 °C until used. All animal experimental protocols followed Yale University institutional guidelines for care and use of laboratory animals and were approved by the Institutional Animal Care and Use Committee (IACUC) at Yale University.

### Genome and RNA sequencing, assembly, structural and functional annotation, and differential gene expression analyses

Detailed protocols for genome and RNA sequencing of *B. microti* isolates are provided in Supplemental Methods.

### Identification of variable mini- and microsatellites, single nucleotide polymorphisms (SNPs) and Nsmall indels

Tandem Repeat Finder (TRF)^59^ was used to identify all micro-satellites and mini-satellites (mx-satellites) in the reference *B. microti* R1genome assembly^25^. In house Perl scripts were used to extract unique sequences flanking the identified mx-satellites. Then, the BLAST^60^ aligner was used to locate each of the unique flanking sequences in other *B. microti* isolate, hence revealing the presence and copy number of mx-satellites homologous to those in the reference R1 genome. In-house Perl scripts were used to determine length and copy number variability of mx-satellites in each isolate compared to reference R1. Bedtools^61^ was used to determine if a microsatellite was present in the exonic, intergenic or intronic region in the genome.

To identify SNPs and small indels, whole genome shotgun sequencing data for each of the strains was aligned to the reference *B. microti* R1 genome^25^ using BWA^62^. Data was formatted using SAM tools^63^ and Picard tools v.1.79 (http://broadinstitute.github.io/picard), and SNP variant calling and filtering using the Genome Analysis Toolkit GATK, UnifiedGenotyper, v2.2.5^64^. In order to remove potential false positives. identified variants were filtered according to the following parameters values: (DP < 12) || (QUAL < 50) || (SB > −0.10) || (MQ0 > = 2 && (MQ0/(1.0 * DP)) > 0.1). SNPs that passed filter were attributed to non-coding or coding regions using VCFannotator (http://sourceforge.net/projects/vcfannotator) in the context of the reference genome re-annotation annotation.

Two approaches were then used to define true variations in the set of *B. microti* genomes. The first variant approach calling using parameters described above provide a list of 1490 possible variation sites where more than 95% were single point mutations. Indels were analyzed differently from SNP. All indels were kept for further analysis whereas the choice of GATK parameters was trained for the choice of the correct filtering threshold. Sanger sequencing of PCR products was performed for six loci: BBM_01g00985, BBM_02g04060, BBM_02g04280, BBM_03g00885, BBM_03g04060, BBM_04g09150. None of the variation described in the vcf files in these regions could confirmed at experimental level. Analysis was done on the 8 strains for loci BBM_02g04280 and BBM_03g04060 and on R1 only for the four other loci. Sequencing confirms the need for a specific and stringent variant calling method. All GATK parameters computed using default option were tested for the signal they provide and correspondence with training mutations. The histogram of several parameter including the ABHom, DP and MQ value was constructed per isolate for each SNP positions. The ABHom parameter evaluating homozygosis at a locus provide valid information over the threshold of 0.85. MQ and DP parameters were also analyzed. We keep SNP position where MQ was over 58. DP had no impact after these two threshold were chosen but in some isolates, DP could be equal to zero in some isolate and this information was identified as uncovered region. We found 889 (588 single point mutations and 301 insertions/deletions) highly reliable variants in the nuclear genome after analysis of eight NGS sequence.

### Strain clustering Analyses based on sequence variation

Unsupervised hierarchical clustering was performed for 7 samples based on 12 variable microsatellites. The pairwise distance between the samples was calculated as the proportion of base substitutions between them over the number of variable microsatellites, *i.e.* for pair of isolates (number of pairwise differences)/(total number of variable sites). Unsupervised hierarchical clustering was also performed based on RNAseq data for 146 differentially expressed genes. Pairwise distance among 6 isolates was calculated as the Euclidean distance. The Ward minimum variance method was used as a metric to build the dendrogram in R for both approaches. Conserved nodes were identified between the two clustering results.

### Immunoproteomic analyses

Detailed protocols for cloning of B. microti cDNAs, microarray fabrication and immunoproteomic analyses are provided in Supplemental Methods.

### Statistical analysis of antibody binding intensity

The data matrix of the compiled intensity data, or “raw” immuno-proteomic data files, were imported in the statistical programing environment R (https://www.r-project.org/). The normalization procedure was as follows: (1) Peak intensity was normalized to the sum of all signals on the array for *B. microti* spots, and (2) intensity of each spot was calibrated to the maximum signal detected in the array. The normalized data (range between 0 and 100%) provide a relative measure of the *B. microti* antigenic response over time compared to day 16 where samples show maximum signal intensity. The data were grouped by time point and sorted by reactivity, and visualized using the RColorbrewer R package to create the color scheme and the gplots R package to generate the heatmap.

### Data Access

Accession numbers for WGS read alignments on reference genome bam files, *de novo* assemblies and RNAseq reads are given in Additional File 2: Table S2. The updated annotation of nuclear chromosomes 1–4 will be associated with features with accession number FO082871, FO082872, LN871598 and LN871598, respectively.

## Additional Information

**How to cite this article**: Silva, J. C. *et al*. Genome-wide diversity and gene expression profiling of *Babesia microti* isolates identify polymorphic genes that mediate host-pathogen interactions. *Sci. Rep.* **6**, 35284; doi: 10.1038/srep35284 (2016).

## Supplementary Material

Supplementary Information

Supplementary Dataset 1

Supplementary Dataset 2

## Figures and Tables

**Table 1:** Distribution of SNPs relative to R1, in seven *B. microti* isolates and in the re-sequenced R1 isolate.

Isolate	Total count^a^	Non coding region			Coding region			Unique SNPs		Ambiguous	
		Inter-genic	Intronic	5′ UTR 3′ UTR	Syn.	Non-syn.	RTH NON	Unique ALT	Unique REF	QUAL	UNC
R1^b^	1	1	0	0	0	0	0	0	515	0	0
ATCC_30222	523	106	49	33	163	170	2	25	0	22	0
ATCC_PRA-99	507	102	53	32	154	164	2	6	0	31	0
GI	528	106	53	33	163	171	2	6	0	7	0
Greenwich_Lab_Strain_1	456	85	44	30	146	149	2	3	0	79	1
Nan_Hs_2011_N11–50	524	104	54	33	164	167	2	5	0	12	0
Naushon	523	107	53	33	162	166	2	3	0	11	0
Bm1438	508	106	48	34	160	158	2	6	0	26	2
**Total number of variable genomic positions**	588	121	63	38	171	193	2	54	515	98	2

^a^Differences were identified against the R1 reference genome^25^.

^b^R1 reference isolate was re-sequenced, using Illumina HiSeq technology, to identify potential errors in the original assembly. The sequence variants identified are based on these Illumina data mapped against the reference genome assembly.

**Table 2:** Distribution of positions with indels relative to R1, in at least one of the seven *B. microti* isolates and in the re-sequenced R1 isolate^a^.

Isolate	Total count	Non-coding region			Coding region			Unique SNPs	
		Inter-genic	Intronic	5′ UTR 3′ UTR	SNP	Indel x3	Other	Unique ALT	Unique REF
R1	35(10)	24(4)	2(1)	0	5	4	0	3	103
ATCC_30222	211(6)	107(1)	58(1)	10(0)	4	17	15	15	0
ATCC_PRA-99	210(7)	111(3)	57(1)	9(0)	3	16	14	1	0
GI	208(5)	112(2)	55(1)	9(0)	2	16	14	6	0
Greenwich_Lab_Strain_1	169(4)	84(3)	47(0)	8(0)	1	14	15	12	6
Nan_Hs_2011_N11-50	221(10)	117(5)	56(1)	9(0)	4	22	13	16	0
Naushon	212(4)	114(1)	52(1)	9(0)	2	16	19	2	0
Bm1438	176(10)	95(4)	54(2)	9(0)	4	13	1	3	1
**Total number of variable genomic positions**	301	160	69	10	NV	28	34	58	110

^a^The table reports all genomic position where at least one strain contains an indel. Other isolates might present single point mutation (SNP) at the same position (shown in brackets). SNP counts were not included in the last row (Total number). NV: Not Valid. Indel x3: indels with length multiple of 3.
