# Supplementary material for: Genome-wide diversity and gene expression profiling of Babesia microti isolates identify polymorphic genes that mediate host-pathogen interactions
Source: Sci Rep. 2016 Oct 18;6:35284. doi: 10.1038/srep35284 (PMC5082761; doi:10.1038/srep35284)
Supplement: Supplementary Information [file srep35284-s1.pdf]

SUPPLEMENTARY INFORMATION

Genome-wide diversity and gene expression profiling of *Babesia microti* isolates identify polymorphic genes that mediate host-pathogen interactions

Joana C. Silva[1,2*], Emmanuel Cornillot[3,4], Carrie McCracken[1], Sahar Usmani-Brown[5,6], Ankit Dwivedi[3,4], Olukemi O. Ifeonu[1], Jonathan Crabtree[1], Hanzel T. Gotia[1], Azan Z. Virji[5], Christelle Reynes[7], Jacques Colinge[4], Vidya Kumar[5], Lauren Lawres[5], Joseph E. Pazzi[8], Jozelyn V. Pablo[8], Chris Hung[8], Jana Brancato[6], Priti Kumari[1], Joshua Orvis[1], Kyle Tretina[1], Marcus Chibucos[1,2], Sandy Ott[1], Lisa Sadzewicz[1], Naomi Sengamalay[1], Amol C. Shetty[1], Qi Su[1], Luke Tallon[1], Claire M. Fraser[1], Roger Frutos[9,10], Douglas M. Molina[8], Peter J. Krause[6] and Choukri Ben Mamoun[5*].

[1]Institute for Genome Sciences, University of Maryland School of Medicine, Baltimore MD 21201. [2]Department of Microbiology and Immunology, University of Maryland School of Medicine, Baltimore MD 21201. [3]Institut de Biologie Computationnelle, IBC, Université de Montpellier, 860 rue St Priest, Bat 5 - CC05019, 34095 Montpellier Cedex 5, France. [4]Institut de Recherche en Cancérologie de Montpellier, IRCM - INSERM U896 & Université de Montpellier & ICM, Institut régional du Cancer Montpellier, Campus Val d'Aurelle, 34298 Montpellier Cedex 5 FRANCE. [5]Department of Internal Medicine, Section of Infectious Diseases, Yale School of Medicine, 15 York St., New Haven, Connecticut, CT 06520 USA. [6]Yale School of Public Health and Yale School of Medicine, 60 College St., New Haven, Connecticut, CT 06520 USA. [7]Institut de Genomique Fonctionnelle, IGF - CNRS UMR 5203, 141 rue de la cardonille, 34094 Montpellier Cedex 05, France. [8] Antigen Discovery Inc., Irvine, CA, 92618 USA. [9]Université de Montpellier, IES, UMR 5214, 860 rue de St Priest, Bt5, 34095 Montpellier, France. [10]CIRAD, UMR 17, Cirad-Ird, TA-A17/G, Campus International de Baillarguet, 34398 Montpellier, France.

* Correspondence to:

Choukri Ben Mamoun: choukri.benmamoun@yale.edu

Joana C. Silva: jcsilva@som.umaryland.edu

A

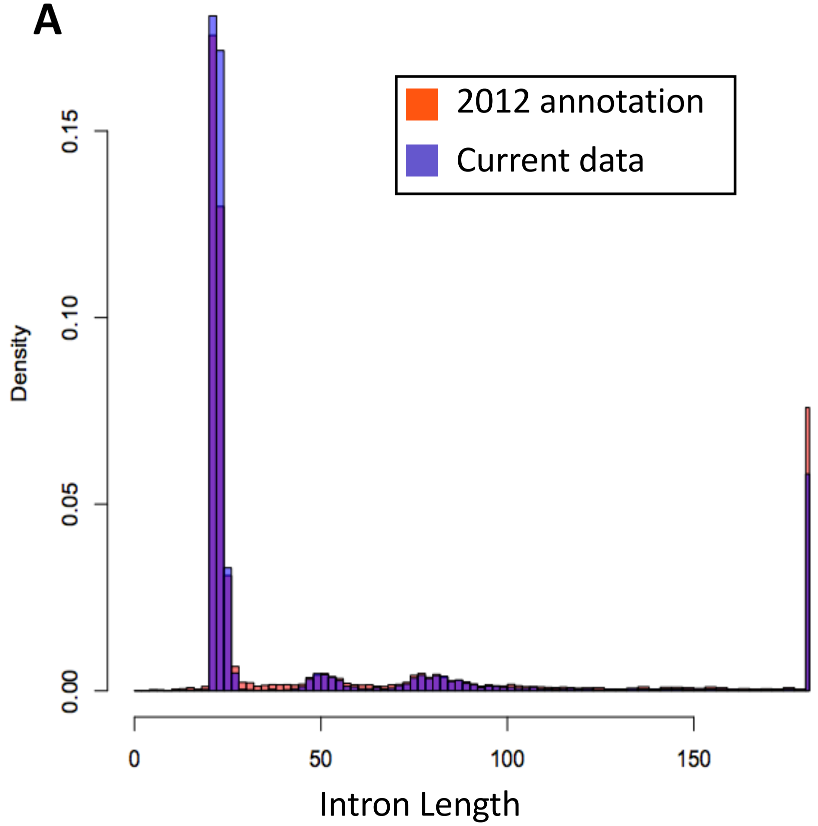

B

Splice sites	Absolute frequency	Percentage
GT...AG	9997	98.6
GC...AG	136	1.3
AT...AC	6	0.1
TG...AG	2	0.0
TG...TA	1	0.0
GT...TG	1	0.0
GT...TA	1	0.0
**Total**	**10144**	

Supplementary Figure 1: Introns statistics. **A)** Distribution of intron size in *B. microti* is multimodal, with mode of 18 nucleotides. The very small modal value already observed in the original ("2012" *Cornillot et al., NAR 2012*) annotation is confirmed and strengthened in the updated ("current data") gene structures. Intron splice sites in the updated gene structure annotation were inferred directly from split reads mapped with Bowtie2. **B)** Frequency distribution of 5´ and 3´ splice site sequences. The canonical GT/AG splice site combination recognized by the major spliceosome is almost exclusively used in *B. microti*.

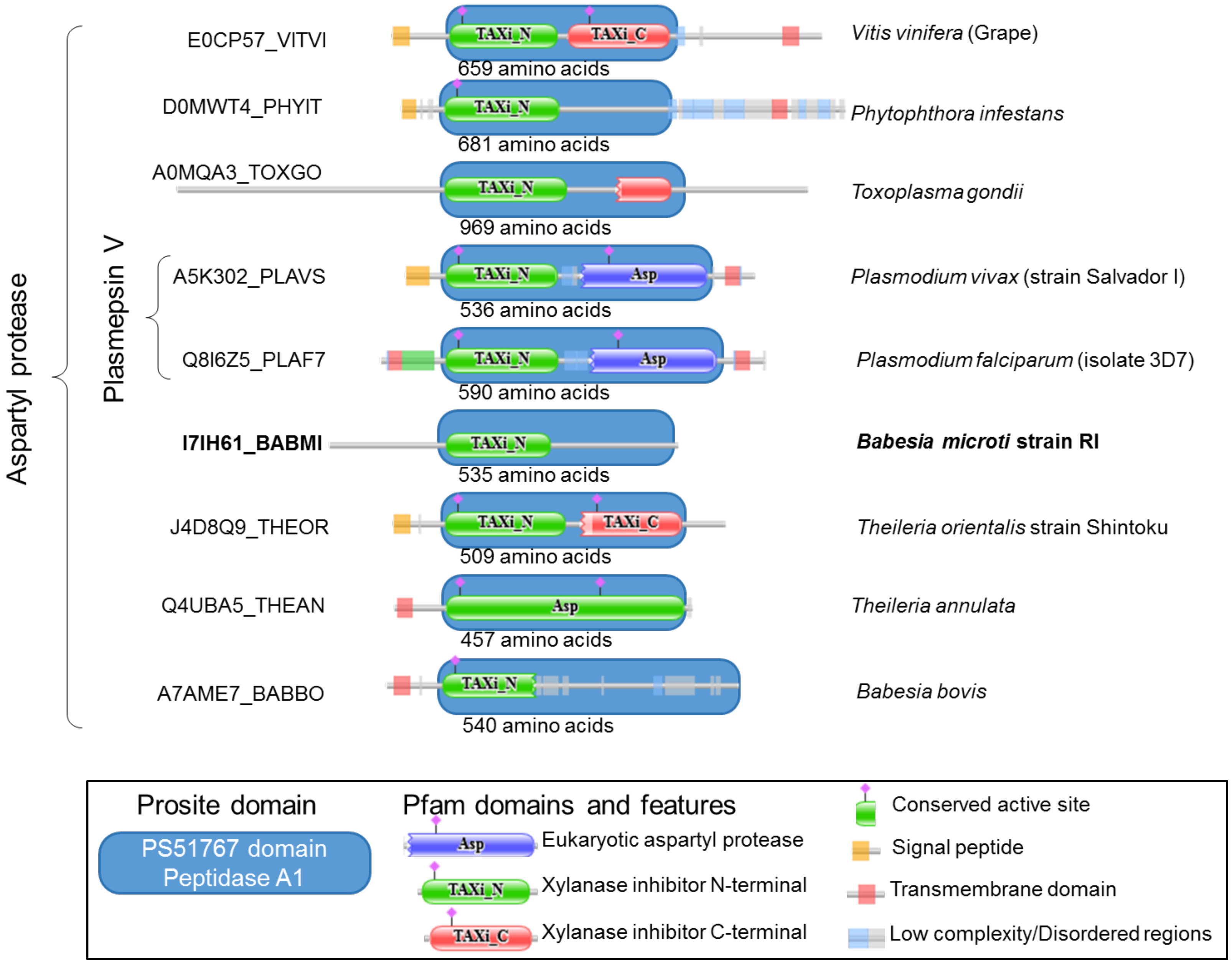

Supplementary Figure 2: Sequence comparison of plasmepsin V orthologs from different apicomplexan parasites. Graphical representation of protein domains was obtained from Pfam database.

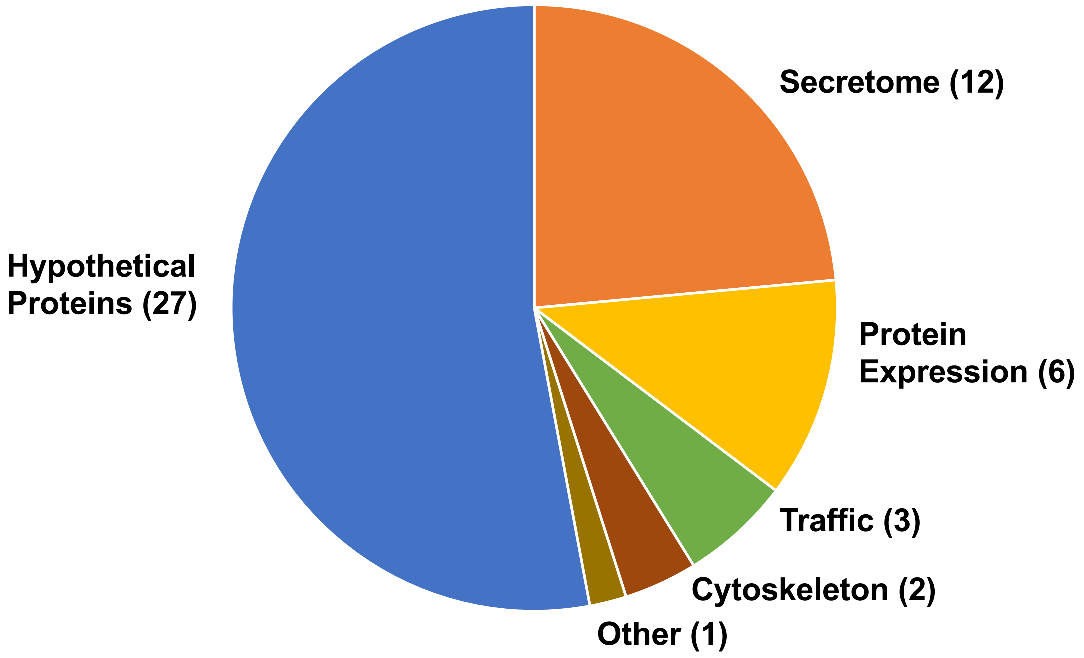

Supplementary Figure 3: Pie chart illustrating the functional distribution of the 50 *B. microti* proteins whose encoding genes are differentially expressed in a host dependent manner.

Supplementary Table S1. Metadata on the *B. microti* isolates used in this study.

	**R1**	**ATCC 30222**	**ATCC PRA-99**	**Bm1438**	**GI**	**Naushon**	**GreenwichYale _Lab_Strain_1 (Lab_Strain_1)**	**Nan_Hs_2011 _N11-50 (N11-50)**
Specimen Source Species	*Homo sapiens*	*Thamnomys surdaster*	*Homo sapiens*	*Homo sapiens*	*Homo sapiens*	*Ixodes scapularis*	*Peromyscus leucopus*	*Homo sapiens*
Species Source Common Name	Human	Forest mouse	Human	Human	Human	Black-legged deer tick	White-footed mouse	Human
Specimen Collection Date	2002	1950	1973	2014	1981	1986	Unknown	2011
Specimen Collection Location	Rennes, France	Congo Kinshasa, Zaire	Nantucket Island, MA	Nantucket Island, MA	Nantucket Island, MA	Naushon Island, MA	Greenwich, CT	Nantucket Island, MA
Laboratory Host (DNA)	Gerbil	Golden Syrian Hamster	C3H SCID mouse	C3H SCID mouse	Golden Syrian Hamster	Golden Syrian Hamster	C3H SCID mouse	Golden Syrian Hamster
Laboratory Host [a] (RNA)	N/A	Golden Syrian Hamster	C3H SCID mouse	N/A	Golden Syrian Hamster	Golden Syrian Hamster	C3H SCID mouse	C3H SCID mouse

[a] The laboratory strain Greenwich and the ATCC-PRA99 isolate can only be propagated in SCID mice. The other four isolates were cultivated in golden Syrian hamsters. The N11-50 strain is the only isolate that could be cultivated in both mammals. In the present study, N11-50 RNA was extracted from parasites grown in a SCID mouse.

Supplementary Table S2. Genome and transcriptome data generated for *B. microti* isolates.

	R1	ATCC 30222	ATCC PRA-99	Bm1438	GI	Naushon	GreenwichYale _Lab_Strain_1 (Lab_Strain_1)	Nan_Hs_2011_ N11-50 (N11-50)
**Genome data**								
gDNA shearing size (bp)	500	241	271	500	240	255	416	303
No. gDNA reads	49,000,000	427,433,106	463,798,222	43,500,000	103,455,108	93,165,064	77,047,792	108,523,562
SRA/ENA accession	ERS1114707	SRP026017	SRP026029	ERS1114706	SRP026012	SRP026015	SRP026028	SRP026025
Genome assembly accession	N/A	JGVA00000000	JGUZ00000000	N/A	JGUY00000000	JGUX00000000	JGUW00000000	JGUV00000000
Assembly length (bp)	N/A	6,630,005	6,346,114	N/A	6,878,190	6,438,007	6,800,559	6,361,046
No. Contigs in assembly	N/A	234	82	N/A	140	131	250	131
Longest contig in assembly	N/A	597,968	619,241	N/A	598,171	366,459	542,955	350,809
Overlaps R1 reference genome (%)	N/A	98.42	98.79	N/A	98.41	98.31	98.90	98.43
**Transcriptome data**								
mRNA shearing size (nucl)	N/A	286	286	N/A	285	286	288	289
No. RNAseq reads	N/A	37,307,396	49,921,782	N/A	43,424,650	48,312,516	52,747,716	56,886,026
SRA accession	N/A	SRS566271	SRS566154	N/A	SRS566158	SRS566212	SRS566229	SRS566414

Supplementary Table S3. Genome features and their properties in *B. microti* and other apicomplexan taxa.

Feature	*B. microti* 2015	*B. microti 2012*	*B. bovis*	*T. parva*	*T. annulata*	*P. falciparum*	*C. parvum*
Genome							
Size (Mbp)	6.4	6.5	8.2	8.3	8.4	23.3	9.1
Number of chromosomes	4	3	4	4	4	14	8
G + C content (%)	36	36	41.5	34.1	32.5	19.3	30.8
Genes							
Number of protein-coding genes	3567	3513	3706	3796	4082	5324	3805
Mean CDS length (bp)	1327	1327	1503	1407	1602	2326	1844
Mean CDS length including introns (bp)	1472	1471	1609	1654	1802	2590	1851
Gene density (bp per coding gene)	1794	1816	2194	2059	2199	4374	2411
Coding regions (%)	74	73	68	68	73	53	76
Coding regions including introns (%)	82	81	73	80	82	59	77
Number of genes with introns (%)	75	70	60	75	71	54	4
Coding Exons							
Number per gene	3.8	3.3	2.8	2.7	3.9	2.6	1.1
Mean length (bp)	347	397	547	514	416	904	1748
Total length (%)	74	73	68	68	73	53	77
Introns							
Number per gene	2.8	2.3	1.7	2.6	2.9	1.6	0.1
Number per gene presenting intron	3.8	3.4	2.9	3.5	4	2.9	1.3
Mean length (bp)	51	61	60	94	70	167	96
Total length (%)	8	8	5	12	9	6	0.02
Intergenic regions							
Mean length (bp)	312	346	585	405	398	1784	561
Total length (%)	18	19	27	20	18	41	23
RNAs							
Number of tRNA genes[c]	44	44	70	71	47	72	45
Number of 5S rRNA genes	2	2	ND	1	3	3	6
Number of 5.8S/18S/28S rRNA units	2	2	5	8	1	13	9

Supplementary Table S4. Accuracy of original annotation, given new annotation.

Feature	R1 Annotation 2012	R1 Annotation 2015	True Predicted	SN[a] (%)	PPV[b] (%)
Nuclear Protein-Coding Genes	3,496	3,567	1,709	48.88	47.90
Coding exons	11,720	13,658	8,712	74.33	63.79
Coding Bases	4,649,316	4,736,816	4,565,742	98.20	96.39

[a] SN: Sensitivity: proportion of true features (genes, exons or coding nucleotides) that were correctly identified in the original annotation.

[b] PPV: Positive Predicted Value: proportion of predicted features that are true in the original annotation, given to the new annotation.

Supplementary Table S5. List of changes in gene structure between original and new annotation of *B. microti* R1 genes.

Number of genes (BmR1-2012)	3,496
Number of predicted genes (BmR1-2015)	3,567
New genes in BmR1-2015	70
Merged genes in BmR1-2015 vs BmR1-2012	107
Number of BmR1-2015 genes overlapping one BmR1-2012 gene	3,335
Number of BmR1-2015 genes overlapping >= 1 BmR1-2012 gene on the opposite strand	14
Number of BmR1-2014 genes completely identical to BmR1-2012 gene (exact intron/exon boundary)	1,709
Number of BmR1-2015 genes overlapping >= 1 BmR1-2014 genes on the opposite strand	0
Missing genes in BmR1-2015	0
Split genes in BmR1-2015 vs BmR1-2012	119
Number of BmR1-2012 genes overlapping one BmR1-2015 gene	3,325
Number of BmR1-2012 genes overlapping >= 1 BmR1-2015 genes on the opposite strand	15
Number of BmR1-2012 genes overlapping >= 1 BmR1-2012 gene on the same strand	22
Number of BmR1-2015 genes overlapping >= 1 BmR1-2015 gene on the same strand	89

Supplementary Table S6. Localization of 262 polymorphic positions relative to R1 reference genome, among seven *B. microti* isolates, with the exception of the 618 fixed differences between R1 and all the other isolates, which are shown in Table S7. These sequence variants include both SNPs and small insertions or deletions (indels). Sequence variationsns are listed in Supplementary Table S9.

Isolate	Total count	Non coding region			coding region				Unique[b]	
		Inter-genic	Intronic	5'UTR 3'UTR	Syn.	Non-syn.	Indel x3	Other	Unique ALT	Unique REF
R1 (Illumina data[a])	27	17	2	0	2	3	3	0	3	Tab. S7
ATCC_30222	130	55	24	4	5	18	10	14	40	0
ATCC_PRA-99	121	56	25	2	2	14	9	13	7	0
GI	116	58	18	2	2	14	9	13	12	0
Greenwich_Lab_Strain_1	78	29	13	2	3	10	7	14	15	6
Nan_Hs_2011_N11-50	130	62	21	2	5	13	15	12	21	0
Naushon	119	61	17	2	3	9	9	18	5	0
Bm1438	89	42	22	2	3	14	6	0	9	1
**Total number of polymorphic sites**	262	117	41	6	10	34	21	33	112	-

[a] R1 reference isolate was resequenced here, using Illumina HiSeq technology, to identify potential errors in the original assembly. The sequence variants identified are based on these Illumina data mapped against the reference genome assembly.

[b] The number of mutations unique to any one *B. microti* isolate is 737 = 112 unique ALT + 618 R1 specific unique REF + 7 other unique REF. The number of phylogenetically informative sites in the *B microti* genome (shared by two or more isolates, but not all) identified from variant calling equals 143, determined as follows: 889 – (Unique 737 + errors in the reference genome 9) = 143. These phylogenetically informative sites consist of 18 SNPs (6 with borderline parameter values) and 125 small indels.

Supplementary Table S7. Localization of 618 fixed differences between R1 and all other isolates.

Isolate	Total count	Non coding region			coding region				Unique	
		Inter-genic	Intronic	5'UTR 3'UTR	Syn.	Non-syn.	Indel x3	Other	Unique ALT	Unique REF
R1	618	156	91	42	161	159	6	3	0	618

Supplementary Methods

Whole Genome Sequencing and assembly. For each isolate, genomic DNA (gDNA) was sheared to average length <500 bp, and used to build a small insert Illumina library. Medium insert libraries were attempted for all isolates, but these DNA samples proved hard to shear to a length of 3 Kb for all samples except Lab_Strain_1. A hybrid 454/Illumina 3 Kb insert library was built by circularizing 3Kb fragments using a 454 library linker and shearing the resulting circularized DNA to ~700 bp. The fragments containing the 454 linker were selected from the resulting fragments and used to build an Illumina library. Both the small and medium insert libraries were sequenced using a MiSeq platform, with each run generating 5-7Gbp in 250bp reads. Raw sequence data is processed through FastQC and other in-house pipelines for sequence assessment and quality control. The sequence data for each sample (except Lab_Strain_1) was assembled with Celera Assembler v7. For the sample Lab_Strain_1, with sequence data from both small and medium insert libraries, was assembled with the Maryland Super-Read Celera Assembler, MaSuRCA. Both assemblers are available through sourceforge. Host DNA contamination was estimated from the proportion of raw sequence reads that mapped to the host genome, and was eliminated from each sample by filtering both reads and contigs against the genome assembly of its respective laboratory host.

Transcriptome sequencing and assembly. A strand-specific cDNA library was built for each sample [1], and RNA-seq data was generated in a Illumina HiSeq2000 sequencer. RNA-seq reads were mapped onto the genome sequence with Bowtie2 [2]. The RNA sequences were also assembled into transcripts, using both *de novo* and genome-guided protocols implemented in Trinity [3]. The transcripts were then mapped to the genome using PASA [4].

The combined data of both ATCC strains, including the alignment of both RNA-seq reads and the joint reconstructed transcriptome, were displayed in Web Apollo [5], to aid in the automated as well as the manual structural annotation of the genome.

Genome annotation. *Ab initio* gene model predictions were created using four gene predictors. GeneMark-ES [6] and FGENESH [7] do not require a training set and were ran directly on the reference *B. microti* R1 assembly [8]. The gene predictors SNAP [9] and Augustus [10] require training with a reliable gene set. A gene training set was created by selecting from the original *B. microti* R1 [11] a subset of genes for which the structural annotation of the coding sequence (CDS) was fully supported by the RNA-seq data mapped to the genome assembly. To this end, genome sequence and annotation were viewed on Web Apollo [5]. A total of 463 genes were selected, originating from all four chromosomes, and of varying numbers of exons per gene, representative of the frequency distribution of exon number per gene in this species. Augustus randomly selected twenty genes from these 463 genes, to form a validation set not used for training, and used the remaining 443, as well as RNA-seq data, as training set. The minimum intron length was set to 16 bp.

Results from these four predictors, transcript assemblies and alignments of other apicomplexan proteins to the genome were used together to generate combined predictions with both Glean and EVM [12]. Proteins from other apicomplexan genomes were obtained from EuPathDb [13] and mapped onto the genome using AAT [14]. For EVM, twenty combinations were run with various weighting schemes. These combinations were compared for sensitivity and positive predictive value (PPV) at the gene, exon, and base level based on the validation gene set not used for training.

Finally, we conducted global manual curation of the entire gene set for the species. In this process, each gene was manually and independently reviewed by two human annotators. Annotators based their decisions on a combination of evidence displayed on Web Apollo which included the original annotation, predictions from all for *ab initio* gene finders, the two EVM runs with the highest values for gene level prediction, without sacrificing sensitivity or PPV at the base or exon level, protein alignments RNA-seq data and transcriptome assemblies. Models were chosen from among the predictions and the original annotation that best fit the RNAseq evidence, or manually altered to fit the RNAseq evidence. Either one or both untranslated regions (5′ and 3′ UTRs) were added when start and end of transcripts were unambiguous. Where there were no RNA seq data, the original annotation in that genomic region was retained. The annotations for tRNAs and rRNAs were retained from the original 2012 genome annotation [11].

Only the R1 genome assembly was annotated and manually curated extensively, as described above. Annotation of the genome assembly of the six new *B. microti* strains was achieved by mapping the set of all gene coding sequences (CDSs) resulting from the re-annotation effort of R1 to each new assembly using GMAP [15]. Custom scripts were used to optimize the mapped annotation, such that multiple fragments of the same gene were merged whenever possible.

Differential gene expression analyses. The reads obtained from the sequencing platforms were fed into the TopHat [2] read alignment tool to be aligned to *Babesia microti* reference genome [8] for each of the sequencing datasets. Up to two mismatches per 30bp segment were allowed, and maximum number of alignments per read was set at 25, above which reads were

removed. The alignment BAM files from TopHat were then utilized to calculate read count across gene using HTSeq [22], which in turn was used as input to the R package DESeq [23]. DESeq was used to normalize the read counts for library size and dispersion followed by tests for differential gene expression between *Babesia* isolates grown in the mice and *vs.* the golden Chinese hamsters. The significant differentially expressed genes were determined using an FDR cut-off ≤ 0.05 and at least two-fold change between conditions.

Isolate	Host	Library size (Reads)	Normalization factor
ATCC_30222	Hamster	1783604	0.6084349
#ATCC_PRA	SCID mouse	3164775	1.1604312
#GI	Hamster	2912954	0.7781063
#Greenwich	SCID mouse	11375612	1.2845630
#Nan N11-50	Hamster	3421921	1.0042571
#Naushon	Hamster	979826	1.4110007

The alignment BAM files from TopHat were utilized to calculate the Reads Per Kilobase of gene per Million mapped reads (RPKM) for each individual gene for each individual sample, using in-house scripts and tools. RPKM allowed us to quantify gene expression from RNA sequencing data by normalizing the read counts by gene length and by number of mapped sequencing reads in each of the samples.

Differential gene expression was then evaluated using two methods. In a first approach, the ratio of the normalized expression level over the median was computed for each gene. A ratio was considered to be significant if >3 (up-regulated) or smaller than 1/3 (down-regulated). A second approach was used to evaluate host differential expression using edgeR and DEseq2 methods. RNAseq data was generated for four isolates grown in golden

Syrian hamster and for two isolates grown in SCID mice. The full set of 3618 genes (including tRNA and rRNA) was screened. 70 genes with less than 4.26 median RPKM were eliminated. Because the sizes of the libraries varied between samples, the “Relative Log Expression” (RLE) normalization method was used. This method is implemented in edgeR and DESeq2 packages. These two methods differ in their estimation of the statistical dispersion per gene. The edgeR method was used with exact test and tagwise estimation of the statistical dispersion, and identified a set of 75 differentially expressed genes. The DEseq2 approach was more lax than DEseq and resulted in a set of 107 differentially expressed genes. Of the 59 genes predicted to be differentially expressed by the two methods, 57 were protein-coding genes. The intersection of the two approaches provides a set of 50 protein-coding genes that are differentially expressed.

Pathway analysis. Functional annotation of gene sets associated with SNPs in *B. microti* genome was performed using InterPro annotation and *P. falciparum* reverse-BLAST homologues. Reverse BLAST analysis was performed using the new version of the *B. microti* genome annotation and PlasmoDB v13.0 of the *P. falciparum* genome. We used the definition of chromosome end and of gene orthology as stated in the first release of the *B. microti* genome [11]. Metabolic charts were obtained from the Malaria Parasite Metabolic Pathway database (http://mpmp.huji.ac.il/). Stage specific gene lists of *P. falciparum* were obtained from PlasmoDB. The information is derived from analysis by Lopez-Barragan et al. [24].

Definition of the secretome and membrane proteome. The secretome of *B. microti* is defined as a set of proteins expected to be secreted outside the infected cell. The membrane proteome consists of proteins predicted to be located in the surface of the parasite and/or the

infected host cell. All analyses were performed with the new version of the annotated *B. microti* genome. The initial step in the prediction of the secretome and membrane proteome involved the use of TargetP (http://www.cbs.dtu.dk/services/TargetP/) and TMHMM (http://www.cbs.dtu.dk/services/TMHMM/) programs. A total of 680 secreted proteins and 196 membrane proteins were identified using this approach. 118 of the 680 proteins of the secretome predicted by TargetP had more than one transmembrane domain. These proteins seem to not require a signal peptide to be targeted to the ER. All predicted proteins were then subjected to additional filters to exclude proteins predicted to be residents of the apicoplast or the mitochondria or targeted to other cellular compartments within the parasite. Using these criteria, we established that the secretome of *B. microti* is composed of 196 proteins and the membrane proteome of 205 proteins. Most of these proteins are hypothetical proteins with the exception of Plasmepsin VII and X, which are predicted to be both in the secretome and the apicoplast proteome.

Intracellular proteins. Proteins involved in cellular or nuclear trafficking were characterized based on annotation and orthology with known sets of *P. falciparum* intracellular proteins. A set of *B. microti* 547 genes encoding intracellular proteins was characterized by orthology against a list of 835 *P. falciparum* intracellular proteins including components of the acidocalcisome, clathrine, COP1, COP2, ER, Exocytosis, Golgi, intracellular membrane, cellular traffic, nuclear pore, nucleus, PTEX, Rab, secretion, vacuolar. The *P. falciparum* gene list was obtained from the Malaria Parasite Metabolic Pathway (MPMP) database (http://mpmp.huji.ac.il/). The orthology between *B. microti* and *P. falciparum* was defined by reverse BLAST analysis and identified 2118 matches. The assignment of a protein to a cell compartment was also based on gene annotation using three sources of information: the

annotation process described above, the former annotation [11], and annotation of *P. falciparum* orthologues.

Mitochondria-targeted proteins. The mitochondrial proteome was characterized using two prediction softwares: TargetP and MitoFates (http://mitf.cbrc.jp/MitoFates/cgi-bin/top.cgi). We also used a list of 276 *P. falciparum* mitochondrial predicted proteins from MPMP. The orthology between *B. microti* and *P. falciparum* identified a list of 206 *B. microti* putative mitochondrial polypeptides. The probability threshold for MitoFates was set at 0.385, resulting in 215 candidate mitochondrial proteins. The initial rate of false positive (FP) prediction was estimated at 16%, inferred from the removal of intracellular proteins. Probability threshold of TargetP mitochondrial score was set at 0.55, in order to filter out sequences with lowest probability score and reliability score at 4 or 5. The total number of accepted proteins after TargetP predictions was reduced from 555 to 395 (removal of lowest probability predictions) and to 295 after removing false positives (25% FP rate). Comparatively, the FP rate was only of 10% among the set of 206 mitochondrial protein predicted from orthology with *P. falciparum*. Based on the intersection among the three predicting methods, 448 proteins were identified. The false negative (FN) rate was evaluated using annotated proteins for which the true localization is known. A total of 107 genes presenting a word related to mitochondria in their annotation, and 35 more genes with known mitochondrial function are part of the final set of true annotated proteins. It appears that 34/142 (25%) were not predicted by any of the methods. This true rate of false negatives is probably lower than 25% if we consider that some of these mitochondrial proteins are known to be directed to the mitochondria without a signal peptide.

Apicoplast targeted proteins. The apicoplast proteome was characterized in a process similar to that described above for the mitochondrial proteome. PATS prediction was used to characterize a first set of putative apicoplast proteins (http://gecco.org.chemie.uni-frankfurt.de/pats/pats-index.php). Annotations revealed that 97 of the 350 proteins were false positives after comparison with the intracellular protein set. Surprisingly, we found four rhoptry-targeted proteins, members of the BMN family of surface antigens, and three GPI-anchored antigens among these 97 proteins. TargetP predicted 125 putative chloroplast protein using plant parameters. Using the same approach as for the mitochondrial genome, 78 proteins with score over 0.51 were retained. Annotations revealed a high proportion of false positive prediction and only 26 were considered as putative apicoplast proteins. Most of them were hypothetical proteins. Four were confirmed by other datasets. We used a list of 281 *P. falciparum* apicoplast proteins to identify 215 *B. microti* possible orthologues that could be putatively targeted to the apicoplast. About 60 of these orthologues were false positive hits. The control set of apicoplast proteins was encoded by 29 genes having words apicoplast or chloroplast in their annotation. The IF2 initiation factor and CPN60 HSP proteins were also added to the list. Eight known apicoplast proteins were not detected by any of the approaches.

cDNA cloning for protein array analysis. RNA was isolated from iRBCs as described previously [11,25] and cDNA was synthesized using the ThermoScript RT-PCR System (Life Technologies) including the optional steps. Coding sequences were amplified from 50 ng cDNA using 40mer oligonucleotide with 20 sequence specific bases and 20 base adapter sequence and 5prime Hot Master Mix (5Prime, Gaithersburg, MD) following the manufacturer's instructions. A step-wise annealing temperature, 53 ^{o}C for 15 seconds followed by 48 ^{o}C for 15 seconds, and the addition of 10% DMSO was used for amplication robustness to accommodate the number of coding sequences being amplified. I*n vivo*

homologous recombination in *E. coli* DH5α cells into a T7 expression vector, pXi was performed as originally described by Davies et al. [26]. DNA was purified, checked on agarose gel and sequenced as described previously [26-43].

Microarray fabrication and serology -- Proteins were expressed using an *E. coli* based cell-free in vitro transcription and translation system, 1 nL of unpurified IVTT reactions were spotted onto 16-pad nitrocellulose coated microscope slides, the resulting printed slides were checked for spotting issues and protein expression and probed with samples as previously described. [26-43]**.**

REFERENCES CITED

1 Parkhomchuk, D. *et al.* Transcriptome analysis by strand-specific sequencing of complementary DNA. *Nucleic acids research* **37**, e123, doi:10.1093/nar/gkp596 (2009).

2 Langmead, B., Trapnell, C., Pop, M. & Salzberg, S. L. Ultrafast and memory-efficient alignment of short DNA sequences to the human genome. *Genome biology* **10**, R25, doi:10.1186/gb-2009-10-3-r25 (2009).

3 Haas, B. J. *et al.* De novo transcript sequence reconstruction from RNA-seq using the Trinity platform for reference generation and analysis. *Nature protocols* **8**, 1494-1512, doi:10.1038/nprot.2013.084 (2013).

4 Haas, B. J. *et al.* Improving the Arabidopsis genome annotation using maximal transcript alignment assemblies. *Nucleic acids research* **31**, 5654-5666 (2003).

5 Lee, E. *et al.* Web Apollo: a web-based genomic annotation editing platform. *Genome biology* **14**, R93, doi:10.1186/gb-2013-14-8-r93 (2013).

6 Lomsadze, A., Ter-Hovhannisyan, V., Chernoff, Y. O. & Borodovsky, M. Gene identification in novel eukaryotic genomes by self-training algorithm. *Nucleic acids research* **33**, 6494-6506, doi:10.1093/nar/gki937 (2005).

7 Salamov, A. A. & Solovyev, V. V. Ab initio gene finding in Drosophila genomic DNA. *Genome research* **10**, 516-522 (2000).

8 Cornillot, E. *et al.* Whole genome mapping and re-organization of the nuclear and mitochondrial genomes of Babesia microti isolates. *PloS one* **8**, e72657, doi:10.1371/journal.pone.0072657 (2013).

9 Zaharia, M. *et al.* Faster and More Accurate Sequence Alignment with SNAP. *arXiv* **1111.5572v1** (2011).

10 Stanke, M., Steinkamp, R., Waack, S. & Morgenstern, B. AUGUSTUS: a web server for gene finding in eukaryotes. *Nucleic acids research* **32**, W309-312, doi:10.1093/nar/gkh379 (2004).

11 Cornillot, E. *et al.* Sequencing of the smallest Apicomplexan genome from the human pathogen Babesia microti. *Nucleic acids research* **40**, 9102-9114, doi:10.1093/nar/gks700 (2012).

12 Haas, B. J. *et al.* Automated eukaryotic gene structure annotation using EVidenceModeler and the Program to Assemble Spliced Alignments. *Genome biology* **9**, R7, doi:10.1186/gb-2008-9-1-r7 (2008).

13 Aurrecoechea, C. *et al.* EuPathDB: the eukaryotic pathogen database. *Nucleic acids research* **41**, D684-691, doi:10.1093/nar/gks1113 (2013).

14 Huang, X., Adams, M. D., Zhou, H. & Kerlavage, A. R. A tool for analyzing and annotating genomic sequences. *Genomics* **46**, 37-45, doi:10.1006/geno.1997.4984 (1997).

15 Wu, T. D. & Watanabe, C. K. GMAP: a genomic mapping and alignment program for mRNA and EST sequences. *Bioinformatics* **21**, 1859-1875, doi:10.1093/bioinformatics/bti310 (2005).

16 Benson, G. Tandem repeats finder: a program to analyze DNA sequences. *Nucleic acids research* **27**, 573-580 (1999).

17 Altschul, S. F., Gish, W., Miller, W., Myers, E. W. & Lipman, D. J. Basic local alignment search tool. *Journal of molecular biology* **215**, 403-410, doi:10.1016/S0022-2836(05)80360-2 (1990).

18 Quinlan, A. R. & Hall, I. M. BEDTools: a flexible suite of utilities for comparing genomic features. *Bioinformatics* **26**, 841-842, doi:10.1093/bioinformatics/btq033 (2010).

19 Li, H. & Durbin, R. Fast and accurate short read alignment with Burrows-Wheeler transform. *Bioinformatics* **25**, 1754-1760, doi:10.1093/bioinformatics/btp324 (2009).

20 Li, H. *et al.* The Sequence Alignment/Map format and SAMtools. *Bioinformatics* **25**, 2078-2079, doi:10.1093/bioinformatics/btp352 (2009).

21 McKenna, A. *et al.* The Genome Analysis Toolkit: a MapReduce framework for analyzing next-generation DNA sequencing data. *Genome research* **20**, 1297-1303, doi:10.1101/gr.107524.110 (2010).

22 Anders, S., Pyl, P. T. & Huber, W. HTSeq — A Python framework to work with high-throughput sequencing data. *BioRxiv* (2014).

23 Anders, S. & Huber, W. Differential expression analysis for sequence count data. *Genome biology* **11**, R106, doi:10.1186/gb-2010-11-10-r106 (2010).

24 Lopez-Barragan, M. J. *et al.* Directional gene expression and antisense transcripts in sexual and asexual stages of Plasmodium falciparum. *BMC genomics* **12**, 587, doi:10.1186/1471-2164-12-587 (2011).

25 Garg, A. *et al.* Sequence and annotation of the apicoplast genome of the human pathogen Babesia microti. *PloS one* **9**, e107939, doi:10.1371/journal.pone.0107939 (2014).

26 Davies, D. H. *et al.* Profiling the humoral immune response to infection by using proteome microarrays: high-throughput vaccine and diagnostic antigen discovery. *Proceedings of the National Academy of Sciences of the United States of America* **102**, 547-552, doi:10.1073/pnas.0408782102 (2005).

27 Driguez, P. *et al.* Protein microarrays for parasite antigen discovery. *Methods in molecular biology* **1201**, 221-233, doi:10.1007/978-1-4939-1438-8_13 (2015).

28 Baum, E. *et al.* Protein microarray analysis of antibody responses to Plasmodium falciparum in western Kenyan highland sites with differing transmission levels. *PloS one* **8**, e82246, doi:10.1371/journal.pone.0082246 (2013).

29 Lessa-Aquino, C. *et al.* Identification of seroreactive proteins of Leptospira interrogans serovar copenhageni using a high-density protein microarray approach. *PLoS neglected tropical diseases* **7**, e2499, doi:10.1371/journal.pntd.0002499 (2013).

30 Gerns Storey, H. L. *et al.* Use of principal components analysis and protein microarray to explore the association of HIV-1-specific IgG responses with disease progression. *AIDS research and human retroviruses* **30**, 37-44, doi:10.1089/AID.2013.0088 (2014).

31 Hermanson, G. *et al.* Measurement of antibody responses to Modified Vaccinia virus Ankara (MVA) and Dryvax((R)) using proteome microarrays and development of recombinant protein ELISAs. *Vaccine* **30**, 614-625, doi:10.1016/j.vaccine.2011.11.021 (2012).

32 Cruz-Fisher, M. I. *et al.* Identification of immunodominant antigens by probing a whole Chlamydia trachomatis open reading frame proteome microarray using sera from immunized mice. *Infection and immunity* **79**, 246-257, doi:10.1128/IAI.00626-10 (2011).

33 Suwannasaen, D. *et al.* Human immune responses to Burkholderia pseudomallei characterized by protein microarray analysis. *The Journal of infectious diseases* **203**, 1002-1011, doi:10.1093/infdis/jiq142 (2011).

34 Magnan, C. N. *et al.* High-throughput prediction of protein antigenicity using protein microarray data. *Bioinformatics* **26**, 2936-2943, doi:10.1093/bioinformatics/btq551 (2010).

35 Mochon, A. B. *et al.* Serological profiling of a Candida albicans protein microarray reveals permanent host-pathogen interplay and stage-specific responses during candidemia. *PLoS pathogens* **6**, e1000827, doi:10.1371/journal.ppat.1000827 (2010).
36 Crompton, P. D. *et al.* A prospective analysis of the Ab response to Plasmodium falciparum before and after a malaria season by protein microarray. *Proceedings of the National Academy of Sciences of the United States of America* **107**, 6958-6963, doi:10.1073/pnas.1001323107 (2010).
37 Felgner, P. L. *et al.* A Burkholderia pseudomallei protein microarray reveals serodiagnostic and cross-reactive antigens. *Proceedings of the National Academy of Sciences of the United States of America* **106**, 13499-13504, doi:10.1073/pnas.0812080106 (2009).
38 Doolan, D. L. *et al.* Profiling humoral immune responses to P. falciparum infection with protein microarrays. *Proteomics* **8**, 4680-4694, doi:10.1002/pmic.200800194 (2008).
39 Beare, P. A. *et al.* Candidate antigens for Q fever serodiagnosis revealed by immunoscreening of a Coxiella burnetii protein microarray. *Clinical and vaccine immunology : CVI* **15**, 1771-1779, doi:10.1128/CVI.00300-08 (2008).
40 Davies, D. H. *et al.* Antibody profiling by proteome microarray reveals the immunogenicity of the attenuated smallpox vaccine modified vaccinia virus ankara is comparable to that of Dryvax. *Journal of virology* **82**, 652-663, doi:10.1128/JVI.01706-07 (2008).
41 Sundaresh, S. *et al.* From protein microarrays to diagnostic antigen discovery: a study of the pathogen Francisella tularensis. *Bioinformatics* **23**, i508-518, doi:10.1093/bioinformatics/btm207 (2007).
42 Eyles, J. E. *et al.* Immunodominant Francisella tularensis antigens identified using proteome microarray. *Proteomics* **7**, 2172-2183, doi:10.1002/pmic.200600985 (2007).
43 Sundaresh, S. *et al.* Identification of humoral immune responses in protein microarrays using DNA microarray data analysis techniques. *Bioinformatics* **22**, 1760-1766, doi:10.1093/bioinformatics/btl162 (2006).
